# Child Guidance
*A paper read before the Society on Wednesday, October 13th, 1937,


**Published:** 1937

**Authors:** R. F. Barbour

**Affiliations:** Director of the Bristol Child Guidance Clinic


### CHILD GUIDANCE.*

BY

R. F. Barbour, M.B., Ch.B., M.R.C.P., D.P.M.,

*Director of the Bristol Child Guidance Clinic.*

---

The term Child Guidance has only been in use for some fifteen years, and was originally coined in the United States. It is particularly appropriate, as the word "guidance" stresses the preventive side of the work. Actually, child guidance forms one part of the bigger subject of psychological medicine; just as pediatrics is the children's department of general medicine, so child guidance is the children's department of psychological medicine. The children seen at a child guidance clinic are those with psychological problems—the difficult and the nervous children, the "naughty" or "high-strung"—that is, children whose behaviour is causing anxiety to their parents or teachers, and who seem unable to fit in to their environment.

You may hesitate, perhaps, at the suggestion that the sphere of medicine should be enlarged to include such problems as stealing, truancy and the like. These, you may feel, are more in the province of the parson or the teacher than in that of the general practitioner, unless there is some very definite underlying organic pathology such as is found in the brain of the general paralytic or post-encephalitic.

* A paper read before the Society on Wednesday, October 13th, 1937,

Perhaps for one moment it may be worth turning aside to look at certain general concepts underlying the whole of psychological medicine. Not so long ago the tendency was to think of Body and Mind as two separate entities and those who were interested in philosophy pondered upon the possibility of interaction between the two. The mind controlled the body and sick minds were only the physician's concern when a person became completely irrational or so anti-social that for the protection of the majority his liberty had to be taken away from him. As far as morals were concerned a "moral faculty" was postulated, and certain people were considered to be born without it or to have it in an underdeveloped form and such people could be certified as moral imbeciles. These concepts have served their time and are being gradually replaced by holistic theories in which the person is considered as an entity in himself. The individual is more than the mere sum of his parts, he has potentialities greater than those of his organs separately considered, just as an assembled motor-car has properties and functions which are not possessed by the mere collection of the component parts as they lie on the stock-room shelves. A person is an integrated unit—a whole—and functions as such. This we fully realize in everyday life when we say certain actions could only have been done by a certain person, they bear his hall-mark, just as a painting bears witness to the personality of the artist in every stroke of the brush. But it is not my intention to discuss the pros and cons of such theories from the philosophic point of view, but rather to stress the need of such common sense theories when it comes to dealing with many of the practical problems of medicine.

We need not believe in interactionism; but our clinical findings when a person has had a fright include dilated pupils, tachycardia and other signs of sympathetic action. Pavlov's experimental work with dogs has only proved what has been known to physicians for centuries, namely, that a person's digestion is affected by his emotional state. The exact form of a person's fears or worries tends to be the result of his previous experiences, but fear in everyone is accompanied by changes in the vegetative nervous system. A person in a panic has a characteristic blood sugar curve. This is a physical change accompanying a mental state, and when such states recur frequently structural organic changes may occur. It is far from clear exactly how these changes are produced, but the recent advances in endocrinology and biochemistry are beginning to throw light on some of these processes. The essential point, however, is that one cannot expect to find a normal physiology if there is an abnormal psychological state. Our medical findings have to be assessed in the setting of the patient's personality, while on the other hand his behaviour has to be considered as the result not only of the person's previous personality, but also of his present physical state. A person's indigestion may be the result of business worries, while his careless driving may be evidence of his physical fatigue. Medicine has to deal with individuals—with people whose ambitions and fears affect their digestion and whose dyspepsia may be a deciding factor in the history of the world. It is necessary to make this broad approach clear, otherwise some of what follows might seem to be outside the scope of general medicine.

Returning to child guidance, the children seen at the clinic fall roughly into four main groups. First, the aggressive and uncontrollable children who are referred for such reasons as "stealing," "wilful damage," "irresponsible." Second, the more introverted type sent because they are sensitive and high-strung, "slow and awkward," "solitary," "lacking in initiative." Third, those who are in difficulties at school, "backward," "lacking in concentration" or those to whom has been applied the old tag "could do better if he tried." Fourth, those where the emotional strain is shown by unusual physiological behaviour such as tics, stammer, enuresis and hyperkinesis—the latter often referred as "St. Vitus's Dance."

The aggressive children tend to predominate, as their behaviour is more likely to give rise to concern to those in authority. The submissive and fearful child is overlooked, as he does not usually worry his parents or teachers to the same extent. These latter are, however, in many ways the more serious problem, as it is from their ranks that so many of the adult neurotics are recruited at a later age. The types of children referred indicate one of the difficulties of child guidance. The referrals* are based not on the actual importance of the problem, but on what the *adults think* is the importance of the problem, Behaviour which in one family would be barely remarked on is in another the topic of much discussion and concern. Referrals are thus to a certain extent an indication of the community's awareness of children's real needs.

In order therefore to assess any given case one has to investigate not only the child but also his environment. We all realize the importance of environment; we ourselves know that when we are with one set of

* Referral = act of referring (*Webster's New International Dictionary*, 1934), in this context it implies patients referred for examination.

people we feel encouraged to put forth of our best, while with others we feel their critical attitude long before any actual comment is made. An adult, moreover, can to a greater or lesser extent choose his own environment, but the child has usually to accept the surroundings in which he finds himself.

The aim of child guidance is to study a child and not a problem and to study that child in his actual setting, any particular problem being regarded as the outcome of the reaction between the child and his environment. In order to carry out such a study efficiently it has been found useful to have a team of three specially trained workers: the doctor, who should have had experience in both adult and child psychopathology; the psychologist, a lay worker and usually a teacher, who estimates the intelligence and educational achievements of the child; the social worker, who deals with the home surroundings, visits the schools and investigates the environmental side of the problem.

Perhaps it would make the procedure clearer if I explained exactly what happens when a child is referred to the clinic. Children are seen only by appointment, so that there is the minimum of waiting for parents and children. Usually the doctor sees the mother first, when she is asked to state the problem as she sees it and to give the relevant details of the child's past history, including such facts as the dates of walking, talking and of acquiring clean habits; inquiry is also made with regard to previous health, adjustment at school, relationship with other members of the family and previous personality. With this outline the social worker then carries on the interview with the mother, paying particular attention to economic difficulties and emotional strain in the home, while the doctor, in another room, is seeing the child. A great deal can be learned from the way the child reacts to the "clinic situation." In the doctor's room there is a large toy cupboard and a blackboard along one wall. Here the child is encouraged to make himself at home, with varying results. Some are shy and timid, others inquisitive. One will sit restlessly twisting her handkerchief, another will at once launch into a long account of her home. One takes a few toys from the shelves and plays constructively, while another is not content until everything is emptied on to the floor. The room and toys are the same for each child, so that the variations in behaviour are the result of the children's differing personalities and may illustrate vividly the problem. Usually it is possible to carry out the physical examination at the first interview, but if it is felt that this will seriously interfere with the child's rapport with the clinic, then it is postponed till a later occasion. The physical examination is a necessary part of the complete investigation, as not infrequently there are physical factors which are influencing the child's psychological attitudes. Special attention is paid to eyesight, speech defects, deafness, evidence of adenoids, left-handedness and chronic infections, any of which may hinder a child's development. The present physical state of the child has to form the basis of future treatment. A few child guidance problems are the direct result of physical conditions, possibly a slightly larger number are secondary to necessary restrictions imposed in order to safeguard the child's health, but a very large number are the outcome of misinterpreted medical instructions. For example, there is the child with an umbilical hernia whose mother was told that "on no account was she to let the child cry," and who faithfully carried out these instructions for two years, with the result that the child is completely spoiled and has merely to threaten to cry in order to get his own way; then there is the parent who knows that his child has a cardiac murmur, and who is afraid to let him play in the street or climb trees even though he has been passed fit for all games and swimming. In these and similar cases an accurate knowledge of what forms of activity may be prejudicial to health is the first step towards drawing up a constructive plan for the future.

The child returns a week later for a psychological examination. He first has a test to estimate his intelligence. This is usually one of the revisions of the original Binet-Simon tests. These tests are obtained in the following way: several thousand children are given a series of things to do, from this is worked out what the average child of any age can do; if another child is then given the same things to do one can compare his results with this previously ascertained standard and say that he is able to do the work that is usually done by, for instance, a seven-year-old child, that is he has a mental age of seven years. Every child has a mental age, and it need have no close correlation with his chronological age. A child who is seven years old may have a mental age of only a few months but might have one of twelve years. The mental age appears to depend on an inborn constitutional factor, and not to be the result of the educational opportunities that the child has had. Further, the ratio of mental age to chronological age (often called the Intelligence Quotient) does not vary greatly throughout life, and it is therefore possible to say at the age of six or seven years, for example, that a boy or girl has a superior intelligence and will probably benefit from a university education, while it is equally possible to say that a child is " dull normal " and it is pointless for him to try for a scholarship to a secondary school. This innate factor of intelligence is now recognized by the educational authorities, and they are gradually re-organizing their schools so that children can be graded according to their mental age and not according to their chronological age.

After the mental age has been assessed a child is given educational tests, usually confined to reading and mental arithmetic. These tests also are standardized according to age. The educational attainments of a child should correspond to his mental age ; that is, if a child has a mental age of ten years then he should be able to read the words and do the sums that are commonly done by a ten-year-old child. The mental age of a child might be likened to the R.A.C. rating of a car, that is its theoretical capacity : the educational attainments would then correspond to the brake-horse-power or actual output.

If a child's school work is below his mental age then he " could do better." Such a child is called " educationally retarded " and will benefit from coaching. The causes of retardation are many, and include absence from or irregular attendance at school, frequent changes of school with different teachers and new methods, or physical handicaps such as deafness, left-handedness or stammering ; and finally there may be emotional barriers which inhibit his learning. In contrast to the " retarded " child is the " dull " child whose school work is up to the level of his mental age, but the latter is eighteen months or more below his chronological age. Such a child appears retarded if his class is graded according to chronological age, but not if according to mental age. When a child is asked to do work above his mental age he tends to show the strain by increased irritability, restless sleep or other evidence of emotional tension. A child's intelligence functions most efficiently when the work he is doing corresponds with his innate ability: a dull child among intelligent children is being subjected to too great a strain, while the bright child in a class of normal children is not usually extended to his full capacity.

In addition to the educational and intelligence tests a child is usually given some performance tests. School work involves an almost continual use of words, and therefore any child who is not good with words is at a disadvantage. A performance test indicates those who are better at doing practical things. Practical and arithmetical ability often go together. It is common for a child who is fluent with words, and therefore capable of expressing him or herself freely, to be given the credit of a better intelligence than is actually the case, while the slower, more practically-minded child, usually has his intelligence under-estimated.

As has been mentioned earlier, children are integrated individuals, and problems in one sphere of life tend to have repercussions in others. The child who cannot get on at school will be an unhappy child at home, every minor illness will be made the most of if it means that he is excused school. A headache which at breakfast will barely allow a boy to lift his head can disappear in ten minutes once he knows he need not go to school. Many children learn at an early age that no attention is paid to such remarks as "I can't do sums," but if they say: "I feel too ill to go to school" then at once their parents are sympathetic. When a girl talks about school in her sleep, or when there is a close correlation between abnormal behaviour and attendance at school, then the school situation will probably merit investigation. Truancy is nearly always the result of the child's inability to do the work and only occasionally is it sheer devilment. If a boy has tried and tried again, and if his genuine efforts are not recognized as such, then rather than face what are to him unfair rows he stays away. The problem of adjustment at school is an important one, for if the child is retarded, and if the behaviour, be it in the form of exaggerated somatic complaints or truancy, is treated by exclusion from school, then the total situation is only in the long run made worse—the child, although perhaps better while at home, loses more time and on return to school is more retarded than before.

The child's physique and intelligence having been investigated and his behaviour in the clinic observed, it is then necessary to consider the different factors in the environment. When attention was first paid to the problems of children it was thought that there would be a close correlation between bad behaviour and poor social circumstances, but this was not found to be the case. Poverty is seldom a major cause of behaviour problems ; it does, however, tend to play an important secondary part, as the child who is emotionally dissatisfied has fewer outlets if his home is poor, while the boy who has plenty of possessions is more likely to find compensatory satisfactions than the one who has no toys. Outbursts of temper in a two-roomed flat may provoke more retaliation than in a big house, where it is possible to isolate the offender till his rage is over.

It is now commonly agreed that the fundamental causes of difficult behaviour lie in faulty relationships between the child and the other members of the family and this explains why frequently only one child in the family becomes a problem. The family situation is essentially different for each child. Take a family of three : the eldest, the first-born, is an only child until the next arrives, she receives the undivided attention of her parents, is proudly shown to grand-parents and neighbours, and each new development is commented on. Number two is born : let us suppose it is another girl, number one is jealous as she has to divide with a rival her parents' affection. The emotional feelings are very similar to those that occur in married life if a third party enters the picture. As number one sees her rival gradually taking over all the things that originally were hers, her dolls, her books, it is barely surprising that she feels insecure. Then number three, a boy, arrives and again the emotional bonds are altered. Number one still gets attention for being the first to do things, the first to read, to stay up late. The baby boy is especially favoured, as a son was wanted after two girls, and if the finances permit of a family of only three, the mother, instinctively, will tend to keep him a baby. But what about number two ? She is nobody's business ! She is bossed about by her older sister and if she dares to touch the baby, even although he takes her toys, she gets into a row. She senses the lack of affection or rather is aware of the different distribution of it and naturally tries to get her share. She will follow her parents about until they tell her to stop or she will keep repeating the question : " You do love me, don't you ? " until the parents become quite irritated and tell her to be quiet. Deep down in every child and in every grown-up there is an ego-side which needs attention, and if it cannot get it in the form of affection it will take it in the form of dislike.

Now our imaginary three children have the same parents and the same home, but their environment, their hopes and fears, their relationships with each other, are fundamentally different.

The position of eldest child appears to be specially vulnerable, as is that of the only child or the weaker of twins, but other positions, such as middle of three, youngest, only girl, or only boy, also give rise to their own special emotional problems. Position in family is important chiefly as a determining factor in the parents' attitude towards the child. The majority of children's as of adults' psychological problems have their roots in the emotional attachments between parents and their children. These are the primary causes of instability, although it may be a precipitating factor at a much later date that brings the problem into the open. Let me state at once that for effective treatment it is not always necessary to recall these early emotional feelings : a strain late in life may reveal an underlying problem, but removal of the strain may restore the *status quo*.

The infant's relationship with his parents is all-important, as it forms the basis of all his future relationships. If a child is to develop normally he must feel secure. A steady foundation is the essential preliminary for all growth—something, so to speak, that one can push against. For the first year the child finds his security in the affection of his mother, later in the routine life and dependability of those around him. Children are very observant, and are quick to sense a hostile or strained atmosphere such as is found in broken homes or second marriages, where there are liable to be conflicting loyalties. The more regular and orderly the child's home life the less likely is he to feel insecure, as the routine of well-established habits is in itself soothing and economizing of energy, for it does not call for frequent decisions on his part. When, however, father says one thing and mother says another, or when permission to stay up late depends not on the facts of the situation but on whether father is tired or not, then at once the child becomes uncertain. Frequently a child is told not to do this or that because "it worries mother," "makes her head worse," or because it "is bad for father's heart"; these reasons are occasionally valid, but more often they are merely a way of side-stepping an issue. Living in some homes is like playing a game of football, with one referee using Association rules and the other Rugby rules—the poor child never knows which code is in force.

The child's first need is security, its second freedom to develop and time, space and material with which to be constructive. One of the more recent discoveries of psychology is that many of the fundamental patterns of life are formed by the time a child is five years old. They may be modified later, but owing to the stress of modern civilization few children or adults get a chance to recreate these early habits. They tend to remain with us throughout life. Personality traits, such as perseverance and courage, are most easily learned before the age of five, a pattern of failure, such as "it is not worth trying" or "no one understands," formed at this age is hard to eradicate. It is not uncommon to be asked to see a lad in his late teens who is a great worry to his parents as he seems to have no ambition, does not know what he wants, and the parents seek advice as to what career he should follow. On taking the history one finds that the parents did not feel the early years were important, and curbed any originality or independence as it was a nuisance in the nursery. As a result he always did as he was told, and when on leaving school there is no longer anyone to direct him he is lost. A parental attitude of this kind can be a more potent moulding force than any material circumstance, and in considering a child's environment one has, therefore, to investigate the moral as well as the financial standards, the attitude of the parents to their children and to each other as much as the social background, in short, any factor outside the child which is influencing his behaviour.

The investigation of the child is completed when the following questions can be answered : What is the child's present state of health ? What is his innate intelligence and does his school work correspond with it ? Was the early development normal ? How does he fit into the family and what kind of a family is it ? How does he adapt himself to failure and success, to difficulties at home and at school ? Then one tries to build up a dynamic picture of the child and see the problem as a useful thing looked at from the child's point of view. Taking a long view, a child may be worse off because of his bad behaviour, but nevertheless there is a reason for it, it serves a purpose at the time. It is like the girl who, uncertain of her ability to adapt herself to married life, develops an hysterical limp on the eve of the ceremony; as she is ill she obviously cannot marry, and the conflict is solved, but at a price.

Turning to treatment, the first step is to decide what endogenous and what exogenous factors are present. In certain cases a simple explanation to the parents of how the symptoms have arisen is adequate. Easing up the pressure of school work or convincing the parent that Jimmie is really fit will meet the needs of some cases. This, however, is often not sufficient, the habit is too engrained or the parents are unable, without outside help, to alter their point of view. Often a child guidance clinic must also function as a parent guidance clinic. Two infants under the age of twelve months have been referred to the clinic and, needless to say, the treatment was directed chiefly towards the parents. Actually in most cases under the age of five the discussion of the problems with the parents is the essential part of treatment. Between the ages of five and ten treatment of both child and parent is equally important. Above the age of ten more and more can be achieved by working with the child alone. One can do a certain amount even if the parents are not co-operative, but in such cases much of the work is likely to be undone between visits.

With children under the age of ten treatment commonly takes the form of "play-therapy." Free use of words and self-expression through them is a relatively late development, so that one perforce has to use the child's normal means of self-expression which is—play. The clinic playroom is a large room with white-washed walls and little furniture. One wall of the room is a blackboard. There are two trays, on legs, filled with sand: a sink and a tap which turns on and off: plasticine, odd bits of wood, a doll's house and a box of assorted lead toys. Here the child is encouraged to be free, he can do or say what he likes provided that he is not deliberately destructive of property or dangerous to other people. He can be critical of members of his family or of other people if he feels like it. Many people are afraid that such free expression will pass over into licence and will become a habit, so that that person's latter state will be worse than his first. There is, however, no more ground for this fear than that the average patient will develop a keloid. As long as there is a conflict inside a person he remains tense, but once he relieves it in action and, so to speak, gets it off his chest, then he settles down. Our emotions tend to be self-limiting in precisely the same way that our physiological cells seem to know when they have grown to the requisite size. It is very interesting to watch a child, at first aimless, restless, unable to settle, gradually working up to some definite act of aggression. He buries a doll which is symbolic of a hated brother and at once, having perpetrated this act, he feels free, his tension goes and he is able to start constructive work. In the playroom the child is free from the limitations of his home and so is able to solve his own conflicts. With certain kinds of problems it is possible to let two or three children work them out together, but usually each child has to be seen separately.

The full investigation and treatment of each case does take a considerable time. The initial interviews with doctor and psychologist are approximately fifty minutes each. The treatment interviews are either twenty-five or fifty minutes, depending upon the type of case and on how quickly the child can adapt himself to the clinic situation. The time may seem long, but on the other hand the results are worth it when looked at from the point of view of child and society. If it is possible to adjust a persistent case of truancy or stealing in a total time of five to six hours it is surely to everybody's advantage. Many cases are referred because those in authority are at their wits' end, and the only alternative is sending the child away in the hope that someone else will have better luck. A child with a so-called "nasty" disposition can effectively break up the peace of a home, and frequent scenes between parents and children tire both sides out in a way that physical illness seldom does. The maladjusted child tends to find a victim, usually someone else in the family, and then this child also suffers, and so several members of the family are affected. While when a problem child is helped the parents' tension lessens, they become less irritable and the whole family situation improves.

It is, however, not only the treatment cases but the supervision cases that I hope will arouse your interest in the future, those in which a true appreciation of the problem permits of its being solved by simple measures. There are two groups of cases which are common in general practice where the psychological factors are not always fully appreciated. First, those children with mild somatic complaints such as headache, feeling faint, indefinite gastric symptoms. If there are no gross physical findings the possibility of school difficulties or strain at home as the cause of prolonging the illness should not be overlooked. The essential questions both here and in all behaviour problems are—Does the child profit by being ill ? What is the situation that he really does not feel able to face ? Children can have anxiety symptoms similar to those of an adult, and I have seen several cases where the "fear of fainting" had been treated for a considerable period as if it was an actual tendency to faint. One usually finds that the fear is linked up with some special situation, such as school prayers or reciting in class.

The second group is formed by the sufferers from chronic conditions where it is easy to overlook the secondary results of our precautionary measures. Being off games is a real handicap to a child ; regular attendance at a clinic at the same hour each week for several months means, in most schools, that it is always the same class that is missed.

When a routine is being drawn up for a boy or girl one should consider his illness in relation to his total personality. It is one thing to order a child to rest for half an hour every day, but if it involves a struggle every time perhaps some modification of it may be worth while ; for if the child lies down only because he has to, he is likely to spend most of the time fuming inwardly and in anything but a restful condition. If a child feels well he wants to be active, and if limitation is necessary then a reasonable explanation should be given to him as well as to his parents, and the moment any increase of freedom is possible it is only fair that he should be told.

The importance of the psychological adjuvants to medicine can hardly be overstressed. The influence of the physician's personality has long been recognized, and it would appear that in the future psychological factors must receive even greater attention. Child Guidance is merely one indication of this trend in medicine. Lord Horder a few weeks ago in a public address said: "Inevitably the doctor's work in the future will be more and more educational and less and less curative. More and more will he deal with physiology and psychology and less and less with pathology. He will spend his time keeping the fit fit rather than trying to make the unfit fit."

